# Detecting Cyber Attacks In-Vehicle Diagnostics Using an Intelligent Multistage Framework

**DOI:** 10.3390/s23187941

**Published:** 2023-09-16

**Authors:** Tasneem A. Awaad, Mohamed Watheq El-Kharashi, Mohamed Taher, Ayman Tawfik

**Affiliations:** 1 Department of Computer and Systems Engineering, Faculty of Engineering, Ain Shams University, Cairo 11517, Egypt; tasneem.awaad@eng.asu.edu.eg (T.A.A.); mohamed.taher@eng.asu.edu.eg (M.T.); 2 Siemens EDA, Cairo 11835, Egypt; 3 Department of Electrical and Computer Engineering, University of Victoria, Victoria, BC V8W 3P6, Canada; 4 Electrical Engineering Department, Ajman University, P.O. Box 346, Ajman 2758, United Arab Emirates; a.tawfik@ajman.ac.ae

**Keywords:** anomaly detection, cyber-physical security, intrusion detection, machine learning, vehicle diagnostics, vehicular security

## Abstract

The advanced technology of vehicles makes them vulnerable to external exploitation. The current trend of research is to impose security measures to protect vehicles from different aspects. One of the main problems that counter Intrusion Detection Systems (IDSs) is the necessity to have a low false acceptance rate (FA) with high detection accuracy without major changes in the vehicle network infrastructure. Furthermore, the location of IDSs can be controversial due to the limitations and concerns of Electronic Control Units (ECUs). Thus, we propose a novel framework of multistage to detect abnormality in vehicle diagnostic data based on specifications of diagnostics and stacking ensemble for various machine learning models. The proposed framework is verified against the KIA SOUL and Seat Leon 2018 datasets. Our IDS is evaluated against point anomaly attacks and period anomaly attacks that have not been used in its training. The results show the superiority of the framework and its robustness with high accuracy of 99.21%, a low false acceptance rate of 0.003%, and a good detection rate (DR) of 99.63% for Seat Leon 2018, and an accuracy of 99.22%, a low false acceptance rate of 0.005%, and good detection rate of 98.59% for KIA SOUL.

## 1. Introduction

Modern vehicles have transcended their traditional role as mere modes of transportation, transforming into sophisticated computerized systems on wheels, featuring advanced sensors, interconnected networks, and software-driven functionalities. They have gained an increasing number of technological functions and features over the last decade, making them smarter and efficient; however, these innovations have simultaneously exposed vehicles to a new realm of concern: cybersecurity threats.

During the mid-20th century, vehicles began incorporating a range of features, including automatic transmissions, power steering, air conditioning, and enhanced safety measures such as seatbelts. Subsequently, digital engine control units (ECUs) were introduced, particularly in the 1970s, allowing for precise control of fuel injection and ignition timing [1]. This innovation played a pivotal role in enhancing fuel efficiency and curbing emissions. Moreover, advanced safety systems, such as Anti-lock Braking Systems (ABS) and airbags, became standard in many vehicles, significantly bolstering both driver and passenger safety. Moving into the 21st century, the integration of infotainment systems, touchscreen displays, and connectivity features became commonplace in the automotive landscape [1].

Today, advanced vehicles have a network of ECUs, as well as sensors and actuators, that carry out one or more jobs, including those that are crucial. Various ECUs have been designed for data processing and communications within the vehicle network. Due to their huge technological transformation, vehicles now are connected to external networks and are considered as linked devices to the Internet of Things (IoT). Typical vehicle architecture contains ECUs from different domains (e.g., body module, powertrain control, chassis and safety, communication control, and head unit). These domains are connected via several communication buses, such as a backbone high-speed Controller Area Network (CAN) [2] or FlexRay [3], whereas ECUs in the same domain are connected via a communication bus so they can communicate directly with each other. Fully electric vehicles have also become more prevalent, with advancements in battery technology extending their range. The evolution of Advanced Driver Assistance systems (ADAS) has led to features such as self-parking, lane-keeping assist, and adaptive cruise control capabilities. Companies such as Tesla have introduced vehicles with advanced autopilot features, moving closer to fully autonomous vehicles. Contemporary vehicles come outfitted with cameras, sensors, and connectivity features for functions such as remote diagnostics, over-the-air updates, and Vehicle-to-Everything (V2X) communication.

### 1.1. Challenges and Security Concerns of Vehicles

Vehicles have historically been developed without considering full security requirements, depending on the presumption that automobiles function autonomously with a lack of communication capabilities. However, incorporating a wireless gateway as a point of entry into the vehicle’s internal network facilitates remote interaction with the vehicle’s firmware, even during active vehicle operation. This enables remote diagnostics, eliminating the necessity for vehicle owners to physically visit a service station for diagnostics. Additionally, firmware updates can be effortlessly applied to numerous vehicles concurrently, circumventing the requirement to individually connect each vehicle via the On-Board Diagnostic (OBD) module and eliminating the need for cable attachment and detachment [4]. Thus, after the technological advancements in the automotive field, vehicles’ ECUs can be exploited physically through an OBD-II port or remotely through short or long wireless connection (e.g., Bluetooth or Telematics Control Unit (TCU)). Zhang et al. illustrated several ways to intrude on a vehicle including usage of the OBD-II port to monitor the traffic between the ECUs [5]. Most of the ECU-controlling threats leverage diagnostic updates, which enable downloading or updating software. Wolf et al. investigated the risks of ransomware attacks on automotive systems [6]. The ransomware may encrypt one of the vehicle’s critical ECUs, rendering it inoperable except if the user pays to unlock it. In 2015, security analysts showcased the capability to remotely exploit the infotainment system of Jeep Cherokee, gaining control of critical functions such as steering and brakes [7]. This incident prompted a massive vehicle recall and highlighted the security risks associated with vehicle connectivity.

Vehicle-to-Vehicle (V2V) communication allows vehicles to share information with nearby counterparts, improving safety and traffic control. However, insecure V2V communication can be exploited by malicious actors. Researchers have shown that a compromised V2V communication system could be used to send false information to nearby vehicles, potentially causing accidents or traffic disruptions [8].

Autonomous Vehicles (AVs) heavily depend on intricate software and sensor systems for safe operation. Any vulnerability or exploitation in this technology could lead to severe safety risks. For example, there have been demonstrations of potential attacks on AVs, such as confusing their perception systems with specially crafted images or signs, which could cause them to make incorrect driving decisions [8].

Remote Keyless Entry (RKE) systems, used for locking and unlocking vehicles, can be vulnerable to relay attacks, where attackers intercept the signal between the key fob and the vehicle to gain unauthorized access. For instance, criminals have used relay attacks to steal high-end vehicles. They intercept the signal from the owner’s key fob and transmit it to the vehicle, allowing them to unlock and start it without physical access to the key [9]. Back in 2016, vulnerabilities were uncovered in Volkswagen (VW) vehicles’ RKE systems, which could potentially allow attackers to unlock VW vehicles [8,10].

As for ecosystem complexity, the modern vehicle ecosystem involves numerous interconnected components, including sensors, ECUs, and in-vehicle networks. The complexity of this ecosystem increases the attack surface. This can be shown in the vulnerabilities discovered in the in-vehicle infotainment systems that have been leveraged to obtain entry to the internal network of a vehicle, potentially compromising critical systems such as brakes and engine control. Demonstrations of vulnerabilities in infotainment systems came to light when the BMW ConnectedDrive infotainment system was compromised [8,11,12]. This was partly due to the fact that its corresponding in-vehicle network gateway, known as the Combox, lacked robust security measures [8,11,12].

As vehicles become more software-driven, the presence of software vulnerabilities can pose significant risks. Notably, Tesla vehicles, known for their over-the-air update capability, have encountered security issues. Researchers revealed that Tesla vehicles can be invaded and it is possible to remotely manipulate critical vehicle systems [13]. Additionally, components and software in vehicles are sourced from various suppliers, and any compromise in the supply chain can introduce vulnerabilities. The automotive industry is still working to establish comprehensive cybersecurity standards and practices, leaving gaps for vulnerabilities and inconsistencies.

### 1.2. Problem Statement

The primary challenge that needs to be addressed is the detection of potential attacks in the in-vehicle network. Therefore, it is essential to determine which components require monitoring and to propose a robust IDS, along with determining its optimal placement.

Attacks can target various vehicle components, encompassing data, in-vehicle hardware, software, framework, and media infrastructure [14]. Specifically regarding data, attackers possess the capability to focus on data stored within specific ECUs [14]. These data may encompass confidential elements such as digital certificates, cryptographic private keys, or particular activities associated with the vehicle and the driver, such as vehicle location and navigation details [14]. In-vehicle hardware attack typically needs to compromise the hardware infrastructure, including car-top units, sensors, and ECUs, necessitates physical access to the targeted devices [14]. An attack on in-vehicle navigation systems can take place through the substitution of a legitimate device with a malicious one or the accidental installation of erroneous hardware as case in Volkswagen’s emission scandal [14]. As in the case of media infrastructure, certain attacks may focus on the vehicle’s surroundings [14]. A common illustration of such an attack involves manipulating electronic road signs. As for software and framework, the presence of a vast array of integrated software within each vehicle, along with differing levels of security control among various vendors, increases susceptibility to potential attacks [14]. The framework responsible for managing ECUs can become a focal point for various forms of attacks.

In order to detect such attacks, it is essential to introduce appropriate IDSs. However, determining their optimal location can be a challenging task. Several factors play a pivotal role in making decisions about deploying IDSs in vehicles. These factors include limitations on ECU power consumption, the necessity to minimize CPU utilization impact, the intricacies involved in modifying the network structure of each manufacturer’s vehicles, and the cost implications associated with deploying more robust ECUs. Therefore, the most advantageous locations for IDS deployment, as mentioned in [15], are within the central gateways, ECUs, and CAN networks. An IDS, known as a host-based IDS, is connected to vehicle ECUs, offering a comprehensive view of internal activities and the capability to detect injected malicious code in real-time. In contrast, network-based IDSs are positioned within the CAN network and central gateways, where they monitor the onboard vehicle network. A prior investigation conducted in [12] delved into the consequences of attacks targeting ECUs situated at distinct positions within the in-vehicle communication system. It was observed that the potential risk associated with a compromised CAN network and central gateways outweighs that of a vulnerable ECU. This arises from the fact that compromised network and central gateways possess the capability to intercept and manipulate data packets as they traverse network gateways leading to particular ECU domains.

Thus, the main motivation for the issues highlighted is to detect potential attacks while also considering the appropriate placement of IDS. Our key focus will be on data attacks capable of disrupting vehicle behavior by identifying malicious diagnostics. This will be achieved through the introduction of a suitable architecture designed to address deployment challenges effectively.

### 1.3. Contributions and the Paper’s Organization

In recent times, different car brands have implemented diverse strategies to enhance their safety measures. For instance, some Original Equipment Manufacturers (OEMs) have introduced access control functions to the OBD-II interface, separated the in-vehicle network into domains, and physically isolated the ECU unit responsible for external communication from the core functions of the vehicle. Additionally, these measures are reinforced by the presence of a firewall to provide further protection.

Our focus in this paper is different than the previous approaches, as it will be concerned with detecting any manipulation in the diagnostic data. The reason for choosing the diagnostic data is that they illustrate and show the state of all ECUs of the vehicle. Usually proposed IDSs focus on detecting malicious CAN messages by detecting any abnormal CAN message frequency or manipulated CAN payloads. Such IDSs need to be located at each ECU to capture the abnormal messages using physical or cyber-physical features. However, some of the introduced IDSs could be inadequate due to the power limitations of ECUs and CAN bus characteristics that need any IDS to process the payload rapidly and without overloading the bus. Diagnostic data are queried and carried by various diagnostic protocols, such as the OBD protocol and the Universal Diagnostic Services (UDS) protocol utilized to transmit information between diagnostic systems and ECUs. In our test case, several diagnostic parameters and Own Parameter Identifiers (PIDs) via the UDS and OBD protocols are collected.

The current trend is to use machine learning and deep learning approaches in the cyber-physical security of the automotive area due to the complexity of attacks. Our work is inspired by different published research papers [16,17,18] that use various deep learning and machine learning models. We extended our previous works [19,20], where the first research introduced a framework (malicious diagnostic detection framework V1) of one layer that uses one machine learning model to detect simple point anomaly attacks in vehicle diagnostics and developed later in the second work, whereas the framework (malicious diagnostic detection framework V2) comprises two layers: the first one is the specification-based detection layer and the second one is the anomaly detection layer that uses Extreme Gradient Boosting (XGBoost) only, which aims to detect complex point anomaly attacks.

Our work’s primary significance lies in its capacity to operate on plain data without necessitating the inclusion of encryption, decryption, or data authentication overhead. Additionally, the proposed architecture is designed to handle various diagnostic data types with minimal concern for the specific protocol or the processing overhead associated with diagnostic message headers, as it operates primarily on logical data. In contrast to our framework, another potential avenue of exploration is the development of strategies for preventing attacks, which is not the primary focus of our framework. The core objective of our framework is the detection of attacks, offering advantages such as real-time monitoring, adaptability to evolving threats, reduced false positives, the capability to identify insider threats, the collection of valuable forensic data for investigations, cost-effectiveness, an additional layer of security, and a commitment to continual security enhancement compared to an exclusive reliance on preventive measures. The significant contributions of our work to the literature are listed as follows:

1. We extended the architecture proposed in work [20], to introduce malicious diagnostic detection framework V3, to not only detect attacks targeting manipulation of signal points randomly (known attacks) but also to detect attacks targeting changes in signals over time (unknown attacks).
2. As for our knowledge, we are the first ones to detect malicious vehicle diagnostic data using the hybrid Generative Adversarial Network (GAN)-XGBoost stacked ensemble technique to solve the gap found in both previous versions. This is carried out by applying an ensemble technique that combines between XGBoost and Gated Recurrent Unit (GRU) GAN to form a base classifier that detects various vehicle diagnostic attacks integrated with the specification detection approach.
3. The performance of the introduced framework has been validated on two different real vehicle data that have different drivers’ behavior, where it achieves superior results compared to different machine learning and deep learning models mentioned in the literature with respect to several metrics.

This research is organized as follows. A brief automotive background about some diagnostic protocols, Diagnostic Trouble Code (DTC), and the used machine learning models in the proposed framework is provided in Section 2. The literature survey will be discussed in Section 3. The proposed framework is illustrated in Section 4. While the datasets, threat, and attack models are declared in Section 5. The results of the framework against different attacks and its performance are discussed in Section 6. The strength points of the proposed framework and its limitations are illustrated in Section 7. The whole work is concluded and future research is discussed in Section 8.

## 2. Preliminaries

This section gives a concise description of DTC and diagnostic protocols, such as UDS and OBD-II. It also offers a brief introduction to the machine learning models employed in the proposed framework, including XGBoost, GAN, GRU, and random forest.

### 2.1. Diagnostic Protocols

OBD-II and UDS are commonly used, so the most substantial information about them will be illustrated in this subsection.

#### 2.1.1. OBD-II

**Figure 1:**
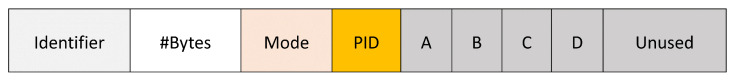
An OBD-II frame’s general structure.

A diagnostic protocol called OBD-II is used to collect emissions information for analysis and monitoring purposes [21]. According to the SAE J1979 standard, each OBD-II PID comprises a code that requests specific data regarding a certain parameter of the vehicle (e.g., engine speed) [22]. Although not all vehicle parameters are covered by OBD-II PIDs, the vehicle manufacturer has the flexibility to modify PIDs utilizing a different diagnostic protocol (e.g., UDS). When a diagnostic tool is attached to the OBD-II port, the ECU responds immediately to the sent OBD-II request. The typical format of an OBD-II frame is shown in Figure 1, where the identification field indicates whether it is a response or request message and the number of bytes field indicates how many bytes are required for each parameter. The OBD-II contains ten modes, some are utilized for retrieving and clearing recorded trouble codes, while others provide real-time information (e.g., RPM). The mode, wherein PIDs are defined, is given in the third field of the OBD-II frame. The relevant PID is contained in the frame’s fourth field. Some PIDs have the formula to convert the relevant PID value to decimal with respect to its minimum and maximum values. The transmitted hexadecimal data bytes that require to be translated into logical values are in the A, B, C, and D fields.

#### 2.1.2. UDS

**Figure 2:**
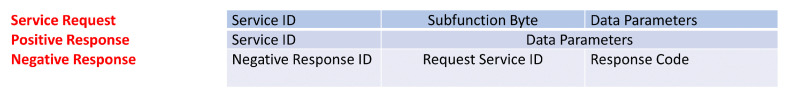
A UDS frame’s general structure.

A diagnostic protocol called UDS could be employed for several communication buses. It provides the flexibility to the vehicle’s manufacturer to define customized PIDs to be analyzed and preprogrammed. The ECU responds to the UDS request with either a negative or positive response using the UDS message format described in Figure 2 to carry out the diagnostic communication over UDS. The Service Identifier (SID) is the first field of the message that is used to differentiate between the request and response messages. Since the Subfunction field is included in certain UDS messages, it is optional. The configuration and additional details of the required parameter are listed in the data parameter field.

### 2.2. DTC

A DTC code is a set of diagnostic trouble codes that are used to notify a problem that occurred by a vehicle’s OBD [23]. Different DTC codes indicate various issues with the vehicle. When the vehicle’s OBD system identifies a problem, it issues a DTC code and sends a warning light to the instrument panel. The alarm can be sent immediately to the fleet in vehicles equipped with a telematics system. The system can be configured to send the alarm to the maintenance department directly.

### 2.3. XGBoost

XGBoost is a distributed, portable, and scalable gradient boosting algorithm [24]. The gradient boosting technique produces predictive models from ensemble weak learners. A typical model in gradient boosting is a decision tree. The trees are constructed sequentially while boosting, whereas each consecutive tree attempts to mitigate the errors of the one before it. As a consequence, an updated version of the residuals will be used to teach the next tree in the sequence. The tree-boosting technique uses *K* additive functions to determine the outputs.

(1) $${\hat{y}}_{i}=⌀\left(X_{i}\right)=\sum_{k=1}^{K}f_{k}\left(X_{i}\right),f_{k}\in F,$$

where F is the tree reconstruction space and $f_{k}$ is among the tree structures whose leaves have $w_{j}$ as a weighted score at each *j*-th leaf. The total of the leaf scores is denoted by *w*. The model must be trained by minimizing an objective function.

(2) $$\begin{array}{c} obj^{\left(t\right)}=\sum_{i}l({\hat{y}}_{i},y_{i})+\sum_{k}\Omega (f_{k}), \end{array}$$

(3) $$\begin{array}{c} \Omega \left(f_{k}\right)=\gamma T+\frac{1}{2}{\lambda ||w||}^{2}, \end{array}$$

where *l* is the differentiable loss function that evaluates the difference between the estimated value $y_{i}$ and the expected value ${\hat{y}}_{i}$, while the regularization function $\Omega (f_{k})$ is employed to avoid model over-fitting by reflecting the tree structure complexity, and *T* represents the number of tree leaves. When the tree cannot be divided any further, the score λ is used, while when an external leaf is introduced, its score is denoted by γ.

### 2.4. GAN

The GAN model is one of the deep learning models that is used to train two simultaneous models [25]. Discriminator (D) and generator (G) are two artificial neural networks that are trained against each other in GANs. The generator model takes random data from the noise vector to generate fake data that will be fed to the discriminator. The generator tries to create data samples that resemble the real data. It learns to generate data by transforming random noise into meaningful data points. Initially, its generated samples are typically random and of poor quality. The main target of the generator is to deceive the discriminator by producing data close to the real data.

The discriminator is a binary classifier that learns to distinguish between false and true data in an unsupervised manner, making GAN a promising technique for anomaly detection. The aim of *G* is to minimize the objective function V(D,G) and *D* tries to maximize it resulting in a two-player min-max game.

(4) $$\begin{array}{c} \underset{G}{min}\;\underset{D}{max}\;V\left(D,G\right)=E_{z∼p_{data}\left(z\right)}\left[\log\;D\left(z\right)\right]+E_{x∼p_{x}\left(x\right)}\left[\log\;\left(1-D\left(G\left(x\right)\right)\right)\right], \end{array}$$

where *z* is a sample from the distribution of actual data $p_{data}\left(z\right)$ and *x* is a sample from the distribution of generator $p_{x}\left(x\right)$.

### 2.5. GRU

GRU belongs to the category of recurrent neural networks [26] and represents a more streamlined alternative to Long Short-Term Memory (LSTM) networks. It exhibits proficiency in handling sequential data, such as text, speech, and time series data, similar to LSTM. At its core, the GRU relies on gating mechanisms to meticulously adjust the network’s hidden state at each chronological step. These gating mechanisms oversee the flow of information within the network, with GRU incorporating two specific gating mechanisms: the reset gate and the update gate. The reset gate’s primary function is to determine the extent to which the previous hidden state should be discounted. Conversely, the update gate plays a central role in determining how much the incoming input influences the modification of the hidden state. The final outcome of the GRU operation is determined based on the refined hidden state.

The following equations can be utilized to compute the update gate, reset gate, and hidden state:

(5) $$z_{t}=sigmoid\left(W_{xz}^{T}x_{t}+W_{hz}^{T}h_{t-1}+b_{z}\right)$$

(6) $$r_{t}=sigmoid\left(W_{xr}^{T}x_{t}+W_{hr}^{T}h_{t-1}+b_{r}\right)$$

(7) $$g_{t}=tanh\left(W_{xg}^{T}+W_{hg}^{T}\left(r_{t}⨂h_{t-1}\right)+b_{g}\right)$$

(8) $$h_{t}=\left(1-z_{t}\right)⨂h_{t-1}+\left(z_{t}\right)⨂g_{t},$$

where $x_{t}$ is the input at time *t*, $z_{t}$ is update gate, $r_{t}$ is the reset gate, $g_{t}$ is the candidate (potential) hidden state, $W^{T}$, *b* are the matrices and vectors of weights and parameters, $h_{t}$ is the current state, and $h_{t-1}$ is the previous (prior) hidden state.

At each time step, the GRU takes in an input vector, which can represent a single element from a sequence, such as a word in a sentence. This input gets combined with the prior hidden state to generate a potential hidden state. The reset gate plays a crucial role in determining which aspects of the previous hidden state are relevant to the current input. It processes both the prior hidden state and the current input, producing an output that ranges from 0 to 1 for each element of the hidden state. Essentially, this gate decides what information from the past should be disregarded or reset. On the other hand, the update gate controls the extent to which new information from the input is integrated into the new hidden state. Similar to the reset gate, it takes inputs from the prior hidden state and the present input, resulting in an output value between 0 and 1. This gate determines how much of the new input should be employed to update the hidden state. The reset gate and the update gate collaboratively compute an interpolation factor, where the reset gate specifies how much of the previous hidden state should be ignored, and the update gate decides how much of the new input should be considered. These gate mechanisms are then used to calculate a weighted average between the prior hidden state and the potential hidden state. The potential hidden state is generated by combining the input with the weighted previous hidden state. In essence, this potential hidden state encapsulates information from both the fresh input and the preceding hidden state. Ultimately, the final hidden state is determined by blending the potential hidden state and the previous hidden state using the update gate. This freshly computed hidden state becomes the output for the current time step and is also employed in subsequent time steps.

### 2.6. Random Forest

Random forest represents a potent ensemble machine learning technique applied in both classification and regression scenarios [27]. Fundamentally grounded in the concept of decision trees, this approach amalgamates numerous decision trees, yielding an ensemble model characterised by heightened robustness and enhanced predictive accuracy. Random forest builds a collection of decision trees during the training phase. Each decision tree is constructed using a random subset of the training data and a random subset of the features (predictor variables). This randomization brings about variability among the trees. Random forest utilizes a method known as bootstrap aggregating, or bagging, which entails the repeated selection of random samples from the training data to generate multiple subsets. A decision tree is then trained on each subset. During prediction, each decision tree in the ensemble provides its output. The final prediction in random forest is determined by combining the outputs of all trees, typically through a majority vote. Decision trees split data at nodes based on criteria such as Gini impurity or entropy for classification. The equations for Gini impurity at node N and Gini gain for a split are as follows.

(9) $$Gini_{N}=1-\sum \left(p_{i}^{2}\right),$$

(10) $$gain=Gini_{N}-\sum \frac{N_{i}}{N}*Gini_{N},$$

where $p_{i}$ is the frequency of a class *i*, *N* is the total count of data samples in the node being considered for split, and $N_{i}$ is the Gini impurity calculated for each child node resulting from the split.

## 3. Literature Survey

Recent studies showed examples of exploiting in-vehicle network messages that can cause deadly failures in the same way that mechanical faults can [28]. The results of investigations into remote attacks on vehicles have surprised manufacturers, resulting in the recall of 1.4 million vehicles [13,29]. Consequently, the cybersecurity aspect of the automotive has been addressed by several recent studies by authenticating the information contained in transmitted messages to thwart attackers from altering them and to check the authenticity of such information [30,31]. Tesla and other electric vehicles are vulnerable to attacks that send bogus State-of-Charge (SoC) information to charging stations in an effort to receive a higher charging priority [32]. Deep learning algorithms are being used in current studies to secure charging stations as well as electric automobiles [33].

Most of the presented IDSs condense on capturing misbehaviour of CAN messages, malicious data in a certain group of sensors, or certain ECU controller behavior. However, most of the IDSs cannot be considered generic systems that can be integrated with any ECU or reliable systems that can be applied. Recent studies used deep learning and machine learning to detect such malicious attacks. For example, Andreas proposed an anomaly detection method that does not require expert criteria to discriminate between unknown and known defects [34]. For univariate and multivariate anomalies, an ensemble classifier is used, which is made up of one-class and two-class classifiers.

For CAN bus traffic, an LSTM neural network-based IDS was suggested [35]. The neural network is taught to anticipate the upcoming values of the data packet, and the errors it produces are used as an indicator to detect anomalies in a series. Other approaches based on regression learning for detecting anomalies in the CAN bus were presented to estimate specific parameters for a certain in-vehicle network attack utilizing correlated/redundant data from a group of sensors [36,37]. A deep neural network (DNN)-based IDS was suggested by Kang et al., where the parameters of the DNN model are trained using probability-based feature vectors collected from the CAN bus messages [18]. Wasicek et al. proposed Context-Aware Intrusion Detection (CAID) IDS for detecting manipulations of a physical system through cyber methods [38]. It employs bottleneck neural networks (NNs) to construct a reference model that depicts the normal behavior of the monitored control system. CAID compares the data to the reference model while it is in operation by executing a plausibility test with the reference model. When the reference behavior and the observed behavior vary, an event is triggered, alerting a possible attack. Seo et al. used GAN and DNN to detect malicious attacks in CAN messages [16]. They used two discriminator models; the first model is trained on known attacks and normal data and the second model is trained on normal data and random data that is generated from GAN. The first discriminator receives the CAN data that are converted into image representation and produces the output between 0 and 1. If the output is lower than the threshold, the input will be classified as abnormal data; otherwise, the data will be sent to the second discriminator. If the output of the second discriminator, which is between 0 and 1 is lower than the threshold, then the data will be classified as abnormal. Furthermore, to extract the spatial and temporal relations in CAN data, Lo et al. developed a deep learning IDS made up of a Convolutional Neural Network (CNN) and LSTM [39]. Preprocessing is performed by their IDS on CAN traffic to minimize inconsistent and missing data. CNN extracts the feature map from preprocessed data. The extracted features are fed to LSTM, which extracts the temporal relationships, and finally, a fully connected neural network is applied, which classifies the output. Basavaraj et al. introduced DNN-based IDS, where the data are encoded and preprocessed before feeding them to the DNN model to detect abnormalities in CAN data [40].

From another perspective rather than all of the above-mentioned work that focuses on IDSs of communication buses, IDSs can be introduced to detect abnormal behavior of in-vehicle diagnostics. Rumez et al. introduced diagnostic Natural Language Processing (NLP)-based IDS. The introduced IDS employs the N-gram approach to detect abnormalities in the sequence of diagnostic messages [41]. The IDS is distributed, where a sniffer in a gateway ECU captures the diagnostic messages on the CAN bus to create a log to be sent to a server for further analysis.

## 4. Introduced Framework

Our framework has multiple stages to be addressed in this section. The problem of IDS location is addressed in our proposed solution. In most cases, IDSs are placed in a gateway ECU or an individual ECU as discussed in Section 1. The drawbacks of employing such an IDS include the power restrictions placed on the ECU, as the IDS should not generally affect the CPU utilization of the ECU, the challenge of modifying the internal structure of each manufacturer’s vehicle network, and the expense of more potent ECUs. To simplify the wiring and typologies of the in-vehicle networks, multiple functions that were previously spread across numerous ECUs are combined into common zone ECUs [42,43]. Such zone ECUs will be linked to one another and convey the entire communications from their smaller subordinate ECUs. Consequently, the zone ECUs should be powerful to perform complex tasks of multiple ECUs. Our proposed framework is introduced to implement an adequate framework that allows one sturdy ECU, which takes the advantage of the zone ECUs, to keep track of the vehicle’s state lowering the expense of using many powerful ECUs.

**Figure 3:**
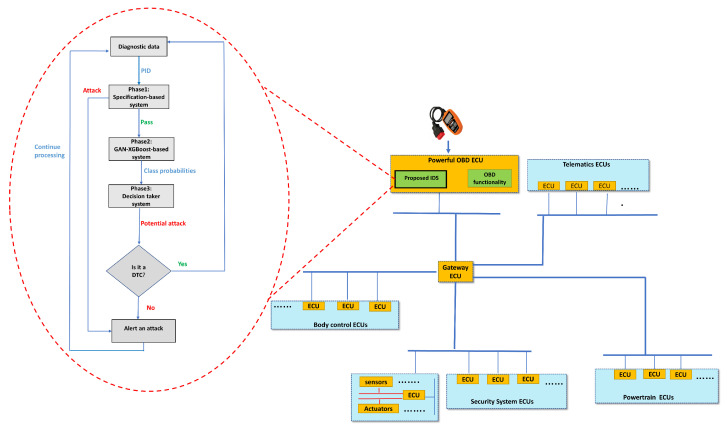
The proposed malicious diagnostic detection framework V3 for the in-vehicle network.

Our framework is located in the end node that is connected to the OBD connector to be a self-invasive system as shown in Figure 3. The framework is deployed in the Open System Interconnection (OSI)’s application layer for generalization. When an ECU with the requisite PID receives a diagnostic request from the framework across the linked bus, it may either reply instantly or divert the message to another ECU that is not directly linked to the end node. After receiving the request, the intended ECU provides the framework with the value of PID. The framework checks the diagnostic parameter based on its specification in the first phase and if the value is abnormal an alert will be raised; otherwise, the diagnostic parameter is sent to the second phase to be processed by XGBoost and GAN models to produce the probabilistic outputs for each class to the third phase. The third stage takes the final decision based on the probabilistic inputs. If the PID is suspected to be malicious, DTC is checked whether an error occurs; otherwise, the aforementioned PID has been manipulated. The actions that could be taken after detecting attacks in the framework could be determined by the vehicle companies’ manufacturers.

Our introduced framework would interpret sending read diagnostic messages while the vehicle is in motion to collect the PIDs as regular communication; otherwise, it will be treated as abnormal messages.

### 4.1. Phase 1

The specification-based system is concerned with detecting any abnormalities regarding the physical constraints of each diagnostic parameter. An example of a used specification rule in our framework is that malicious values of one PID can be captured if they are out of PID’s minimum and maximum ranges. The specification rules can be augmented and updated to adapt each PID’s characteristics. If a malicious attack has been detected in this phase, an alert is raised, and the framework resumes its functionality. However, if the diagnostic parameter is benign at this level, it is fed forward to phase 2 to undergo anomaly attack analysis.

Identifying malicious values in vehicles using a specification-based detection system offers distinct advantages and disadvantages within the automotive context. On the positive side, this system excels at detecting known attack patterns, aligning with the critical need for precision in automotive security. They typically have a low false positive rate at detecting abnormal vehicle behavior that does not conform to the provided specifications, a critical factor in the automotive context where false alarms can result in unwarranted safety concerns or vehicle shutdowns. They can be tailored to specific vehicle makes and models, ensuring a customized approach. Referring to the given example earlier, the system is simple in implementation and expedites the detection process when the diagnostic parameter value falls outside its valid physical range.

However, their limitations include an inability to detect anomaly attacks and the constant maintenance required to update specifications. These systems may have difficulty adapting to changes in vehicle behavior that are legitimate but not accounted for in the specifications. For example, system updates or variations in driving conditions may trigger false alarms. Specification-based systems may lack context about the overall vehicle state, making it challenging to differentiate between legitimate deviations from specifications and actual attacks. False negatives, the critical safety implications associated with them, and the potential overreliance on known threats highlight the imperative for a comprehensive cybersecurity strategy. This strategy should encompass the integration of specification-based systems with other intrusion detection methods to fortify overall vehicle security. Consequently, introducing Phase 2 and Phase 3 of the proposed framework is essential to bolster the detection of anomaly-based attacks, thus strengthening the vehicle’s defense against evolving threats.

### 4.2. Phase 2

The framework’s second phase comprises two models; the first one is the XGBoost and the second one is the GAN model. Both models are combined to take advantage of each other to reach the ultimate accuracy in detection. The XGBoost model is chosen based on its superior empirical results that were shown in previous versions of the framework [19,20] in the detection of known attacks. Furthermore, XGBoost possesses the capacity to handle outliers effectively and incorporates regularization methods such as L1 and L2 regularization [44]. These regularization techniques serve to mitigate the risk of overfitting to noisy data points that lie outside the typical data distribution. GAN is one of the models that is used for anomaly detection using normal data as the abnormalities can be captured by finding a high reconstruction error between the input data and the predicted data [25].

To achieve optimal performance within our framework, it is essential to configure different hyperparameters for each model. In our case, we focus on fine-tuning the hyperparameters of the XGBoost, GAN, and random forest algorithms. The critical parameters of XGBoost encompass the learning rate, the maximum depth of trees, the ratio of training samples, the minimum reduction in loss, and the minimum child weight [45]. In one study [45], the optimization process employed Randomized Search CV, a technique that selects a predetermined number of hyperparameter combinations from a specified search space. This approach utilizes cross-validation to comprehensively evaluate the performance of each hyperparameter combination. In another investigation conducted by Ahmed et al. [44], XGBoost hyperparameters were optimized using Bayesian optimization with cross-validation. This technique was preferred due to its superior efficiency when compared to the grid search method. The parameters subjected to optimization included the learning rate, the maximum tree depth, the rates of L1 and L2 regularization, the minimum reduction loss, and the minimum child weight [44]. Furthermore, an alternative optimization strategy was employed, leveraging a genetic algorithm (GA), as demonstrated in the work [46]. This study illustrated how GA enhances the search algorithm by drawing inspiration from the principles of natural evolution. As a result, these parameters were effectively tuned to enhance model performance.

In our context, we adopted the Nondominated Sorting Genetic Algorithm II (NSGA-II) [47] to optimize the parameters of each model in our test cases [20]. This comprehensive approach allowed us to obtain the most favorable parameter values. Specifically, we utilized NSGA-II to fine-tune the aforementioned critical XGBoost parameters, along with other parameters that significantly influence model performance, such as the learning rate, the maximum tree depth, the training sample ratio, the sampling method, the minimum reduction loss, and the minimum child weight. This meticulous parameter optimization process was conducted to achieve superior performance in our detection tasks. The range of values explored in our research for hyperparameters of XGBoost can be found as follows:
eta ={0.10, 0.15, 0.20, 0.25, 0.30, 0.50, 0.60, 0.70, 0.90}maximum depth ={6, 8, 10, 12, 15, 20, 30}minimum child weight={1, 3, 5, 7, 9}gamma ={0, 0.1, 0.2, 0.3, 0.4, 0.5, 0.7, 0.9}subsample ={0.1, 0.3, 0.5, 0.7, 0.9}sampling method ={uniform, gradient based}

To prevent overfitting in our case, the learning rate value is 0.1, and to prevent the model’s complexity, the tree’s maximum depth is 15, while the ratio of training samples is 0.9 using a uniform sampling method. The minimum reduction loss value to carry out the further leaf node split is 0. The value of the minimum child weight of the instance to stop the partitioning process is 3.

The data used in this research are a continuous time series, so we used a variant of GAN which is based on GRU to capture the temporal dependencies and hidden time-related patterns for each diagnostic parameter. The reason behind choosing the GRU network in GAN over other neural network architectures, particularly CNN and LSTM, in the discriminator and generator is to capture the temporal dependencies in the data and reduce the risk of vanishing gradients compared to vanilla recurrent neural networks, while being computationally efficient. LSTM can detect complex temporal relationships and long-term dependencies; however, GRU is preferred in this research, as it has a lower number of parameters, computational efficiency, simpler architecture, faster training, and the ability to mitigate overfitting. Conversely, CNNs are better suited for tasks involving grid-like data such as images, emphasizing spatial relationships. Thus, based on the nature data used in this research, GRU is the most suitable architecture for GAN.

Opting for the GRU architecture in both the discriminator and generator, while assigning distinct hyperparameter values to each component, serves the purpose of balancing model symmetry and fine-tuning for their respective functions. Utilizing a shared foundational architecture ensures consistency and symmetry in the network’s core structure, enabling them to benefit from common learning mechanisms. Nevertheless, individualized hyperparameter values, such as the activation functions, number of neurons, and dropout rates, are introduced to customize the behavior of each component to suit its unique task requirements. The GRU discriminator is trained to reduce the mean negative cross entropy between the estimates and training labels, where the discriminator loss can be formulated as follows:

(11) $$D_{loss}=\frac{1}{n}\sum_{i=1}^{n}\left[\log\;D_{gru}\left(z_{i}\right)+\log\left(1-D_{gru}\left(G_{gru}\left(x_{i}\right)\right)\right)\right],$$

where the training samples are formulated in $z_{i},i=1,\ldots ,n$ and $G_{gru}\left(x_{i}\right),i=1,\ldots ,n$ is the generated samples from GRU generator. The generator loss can be modeled as follows:

(12) $$G_{loss}=\frac{1}{n}\sum_{i=1}^{n}\left[\log\left(1-D_{gru}\left(G_{gru}\left(x_{i}\right)\right)\right)\right]$$

In our case, the diagnostic parameters are considered as N-multivariate time series data, where the N-dimensional time series can be modelled as *Z* =$z^{t}$,t=1,…,T, where $Z^{t}$∈$R^{N}$ is the N-dimensional vector of inputs for *N* features at *t*. Consequently, $Z^{real}$ is considered normal data that are used to train GAN and to generate $Z^{fake}$ as fake data to deceive the discriminator. In testing, the $Z^{tes}$ is fed as test data to be analyzed by the trained model.

For GAN, learning rate, activation functions, number of layers, type of optimizer, type of loss function, and dropout value parameters are optimized using NSGA-II. The learning rate affects training stability; the choice of activation functions impacts network capacity and stability; the number of layers influences the network’s capacity and risk of overfitting; the optimizer affects training speed and convergence; the type of loss function impacts training stability and sample quality; and the dropout value is essential for preventing overfitting. Careful tuning of these hyperparameters is necessary to achieve stable and high-quality GAN training, as GANs are known to be sensitive to their settings. The search space of the hyperparameters explored in this study for the GAN can be summarized as follows:
number of neurons ={16, 32, 64, 128, 256, 512, 1024}number of layers ={2, 3, 4, 5, 6}activation function ={relu, softplus, linear, sigmoid, tanh, softsign, softmax}optimizer ={rmsprop, adam, sgd, adagrad, adadelta, adama, nadam}lr ={0.1, 0.01, 0.001, 0.0001}loss ={binary cross-entropy, mean squared error}dropout ={0, 0.2, 0.3, 0.5}

The discriminator after tuning has three layers with 256 neurons for the first two layers. The activation function used in the output layer is softmax, while the rest of the activation functions are sigmoid. The generator has three layers with an activation function Rectified Linear Unit (ReLU) and 16 neurons for the first and second layers. ADAM optimizer is used for updating the parameters. To avoid overfitting, regularization methods such as L2 are used to control the complexity of the model besides to dropout layer inserted after the second layer to drop some neurons with the value of 0.5 and the used loss function is binary cross-entropy. The stopping criterion is set at 12; nonetheless, the number of iterations differs among vehicles. For instance, it typically reaches 2050 iterations for the Seat Leon 2018 and 2750 iterations for the KIA SOUL.

**Figure 4:**
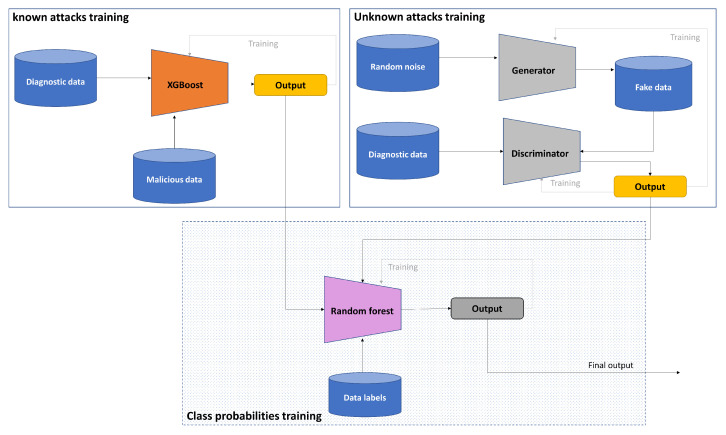
The hybrid GAN-XGBoost stacking ensemble training process within our framework.

The training procedure of our framework is shown in Figure 4, where the GAN model trains on normal data exclusively for identifying unknown attacks, while the XGBoost trains on labeled datasets comprising malicious and benign data. The predicted class probabilities of each class from the two models after the training process are the input to the third phase in our framework, which makes the decision.

In NSGA-II, we utilized a population size of 20 and ran the algorithm for 15 generations. The rates of crossover and mutation applied were both set to 0.9. In our scenario, the problem population comprises various models of one kind of machine learning models used in the proposed framework, each with distinct model values. We treat each model parameter as a gene, building upon our prior work [20]. In our problem, the chromosome representation takes the form of a vector consisting of sequential model parameters [20].

### 4.3. Phase 3

A simple random forest classifier is used in the last phase as a meta-classifier to produce the final decision from the ensemble of the two classifiers. The use of the random forest is to avoid the problem of choosing the suitable threshold, which may take much time to obtain such information empirically. Random forest was chosen as a meta-classifier in stacking ensemble, since it is simpler to use and requires less parameter tuning, and it also provides diversity, resilience to overfitting, and efficiency when aggregating predictions from a variety of base learners. The following parameters have been optimized to achieve specific values: the random forest consists of ten trees, the splitting criterion is based on Gini impurity, the square root function determines the maximum features, and the maximum depth of the trees is set to 6. Additionally, the minimum weighted fraction required for a leaf node is defined as zero, based on the combined weights of all input samples. The number of trees influences robustness, with more trees generally reducing overfitting. The choice of splitting criterion affects tree structure and, consequently, model performance. The maximum features parameter balances model diversity. Limiting the maximum depth of trees prevents overfitting but risks underfitting if set too low. The minimum weighted fraction for leaf nodes controls tree growth and overfitting potential. The search space of hyperparameters of random forest can be listed as follows:
criterion ={entropy, gini}maximum depth ={6, 10, 20}maximum features ={sqrt, log2}minimum weight fraction leaf ={0, 0.3, 0.5}number of trees ={5, 10, 20, 50, 100, 200, 300}

**Figure 5:**
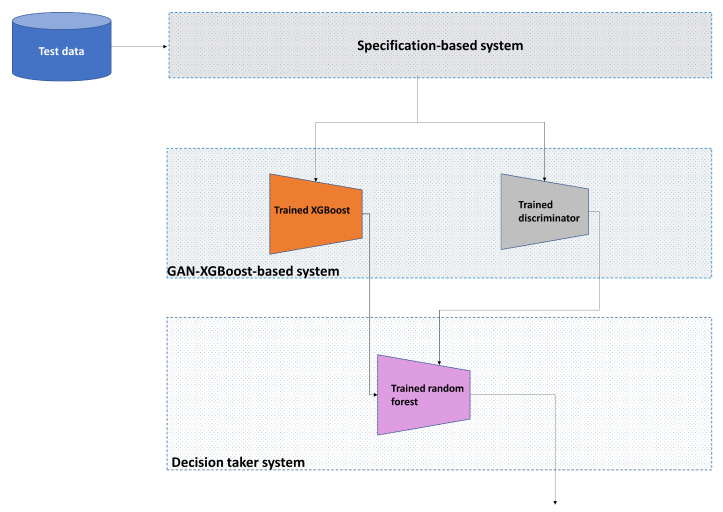
Our framework identification process.

The input to the classifier is the probabilities of classes, which are generated from XGBoost and GAN models, and class labels to indicate whether the class is benign or malicious in the training process as shown in Figure 4. In the testing phase, the random forest processes only the probabilities of the classes that are produced from trained XGBoost and trained GRU discriminator, to provide a binary decision on whether the processed PID is benign or malicious, as illustrated in Figure 5.

Our objective is to guarantee that we can detect unexpected attacks with high accuracy. The primary rationale for exclusively training the discriminator (GRU model) on normal data only is to increase the likelihood of detecting unknown attacks as anomalies, as they deviate from the learned normal behavior. This approach is chosen because if the model is trained using a mixture of normal and attack data, it may encounter difficulties when trying to generalize to previously unseen attacks, given that it has acquired a discriminatory bias against familiar attack patterns. Nevertheless, employing the discriminator learned with only benign data can result in lower detection accuracy than using the XGBoost trained with the attack data. As a result, we integrate the XGBoost and the discriminator resulting in a system that can identify both known and unknown intrusions. This conclusion is derived from the analysis of the results presented in Section 6.

Generally speaking, utilizing anomaly-based IDSs to identify malicious values presents both notable benefits and drawbacks. On a positive note, these systems demonstrate a strong capability for uncovering previously uncharted and innovative threats, showcasing adaptability in the face of evolving attack methods and scenarios, and delivering timely alerts for emerging concerns. These systems can adapt to changing environments and evolving attack techniques because they continuously learn and update their understanding of what constitutes "normal" behavior. Anomaly IDSs can consider the context of events, helping to reduce false positives by taking into account whether a deviation is genuinely suspicious given the circumstances. In contrast, such IDSs can be complex to set up and fine-tune, requiring a substantial amount of data and time to build accurate baselines of normal behavior. Determining what constitutes "normal" behavior can be challenging, and it can vary depending on the context, making it harder to implement effectively. Hence, the development of a robust anomaly-based detection system with efficient computational capabilities is crucial to minimize false positives and prevent performance burdens on the vehicle system.

Finally, determining whether a detected issue in a vehicle is a suspicious attack or a legitimate DTC involves various considerations. Ensuring that detected issues are not malicious attacks but rather genuine diagnostic trouble codes helps prevent unnecessary safety concerns or disruptions in vehicle operation. Correctly identifying issues as DTCs reduces false alarms and minimizes the risk of overreacting to benign vehicle behavior. As for its negative side, misclassifying an attack as a DTC could lead to a delayed response to a security breach, potentially allowing attackers to exploit vulnerabilities and compromise vehicle safety. If an issue is incorrectly classified as a DTC when it is, in fact, an attack, it could pose serious security risks to the vehicle and its occupants.

## 5. Datasets and Attack Design

This section discusses the datasets, threat, and attack models used in this research.

### 5.1. Dataset

Our work is evaluated on two datasets acquired from the KIA SOUL [48] and Seat Leon 2018 [49] vehicles. The Seat Leon 2018 dataset is available online on the website of Karlsruhe Institute of Technology (KIT) [49]. The dataset was collected through OBD-II every 0.1 s. It contains ten diagnostic parameters, while the KIA SOUL dataset contains 51 diagnostic parameters. KIA SOUL dataset is accessible online on the website of Hacking and Countermeasure Research Lab (HCRL) [48]. The data are captured through OBD-II every 1 s. The total number of records in KIA SOUL is 94,401 for ten drivers, while it is 52,938 for Seat Leon 2018. Since the features have various scales, they are normalized before feeding them into the GAN-XGBoost-based system as the deep learning model is sensitive to heterogeneous features, which have different scale values. The datasets have a two-dimensional shape, with one dimension reflecting the values of the signals across time and the other comprising diverse PIDs, aligning with the format of our framework.

The majority of IDSs mentioned in the literature outlined model attack scenarios for CAN messages, such as a denial of service attack, as well as a masquerade attack, a replay attack, and injection of spoofing fuzzy messages without disclosing the used datasets in their research. For CAN communications, malicious and benign datasets were offered by other works [50,51]. Nonetheless, we were unable to operate on them because their representation varies from the structure required by our application layer architecture. For example, each row of the “Car Hacking: Attack Dataset” represents the physical values of each in-vehicle signal associated with other fields, such as the number of data bytes, CAN message ID, and time stamp [50].

**Figure 6:**
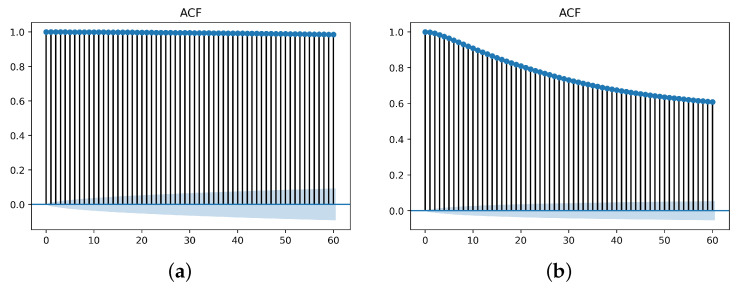
ACF graphs of the vehicle speed of the KIA SOUL and Seat Leon 2018 datasets. The ACF plot displays the correlation coefficient on the y-axis and the number of lags on the x-axis. (**a**) The Seat Leon 2018 dataset’s ACF graph of a vehicle speed signal. (**b**) The KIA SOUL dataset’s ACF graph of a vehicle speed signal.

Figure 6 shows the periodic behavior of vehicle speed in the given datasets using the Autocorrelation Function (ACF). ACF illustrates the relation of the current value of the signal with its past values. If at any instant the signal is bounded by the blue shaded area, which is the confidence interval, then the signal becomes less correlated with its previous values. Usage of such a characteristic helps machine learning and deep learning models to capture easily the deviation in the behavior when an attack occurs.

### 5.2. Threat and Attack Models

**Figure 7:**
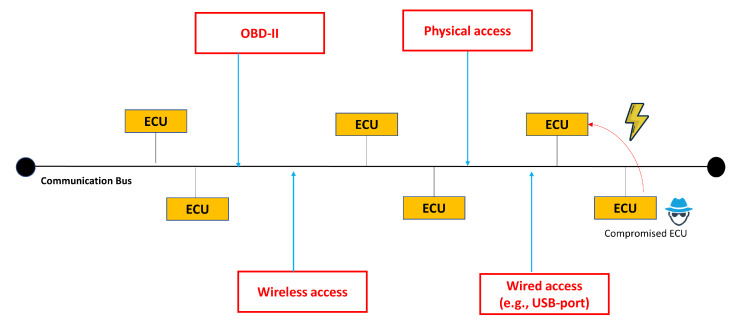
Example of threats that can be exploited to attack a vehicle network.

Figure 7 shows an example of the vulnerabilities that can be exploited to disturb the communication network of the vehicle in our system. The internal automotive system can be invaded through physical access, OBD-II, wireless access, or wired access, which results in the modification of diagnostic parameters in a memory. Another scenario is where an infected ECU can manipulate the values of diagnostic data in other benign ECU.

There are different types of attacks, including attacks that impact the timing behavior of a signal and attacks that manipulate the value of a signal. We focus in this research on point anomaly and period anomaly attacks. A point anomaly attack targets manipulating a random data point of a feature at a random time. Conversely, period anomaly attack targets manipulating data points over a period of time to deviate the time series behavior of a PID. Those types of attacks can generate scenarios of replay, masquerade, and chip-tuning attacks. We choose to evaluate point anomaly attacks and period anomaly attacks in the context of cyber-physical security due to their representation of real-world scenarios, the diversity they offer in attack types, and the unique detection challenges they pose. These attacks help assess the robustness and adaptability of security solutions, considering the different impacts they may have on critical infrastructure systems. Point anomalies, such as sudden sensor anomalies indicating potential sensor tampering or malware injection, and period anomalies, such as consistent manipulation of GPS data over time, serve as realistic threat models.

The malicious data used in this research are generated by applying the point attack models shown in Table 1 that were proposed in our previous work [20] for supervising the training of the XGBoost model. At a selective time, one of the attack models manipulates the benign data. In Attack A, the intruder tries to manipulate a particular PID at a certain time by lessening the value with a random negative or positive value. In Attack B, the intruder tries to deceive the neighboring ECUs by sending a previously manipulated value of a certain PID at a specific time. In Attack C, all PIDs can be set to zeros to insert disorderliness in the system. While in Attack D, two random PIDs at a certain time are manipulated with different random values.

**Figure 8:**
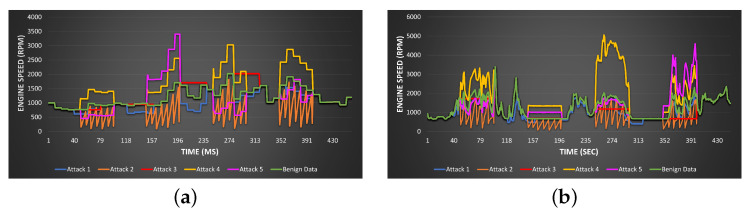
Benign engine speed versus malicious engine speed generated from Attack 1, Attack 2, Attack 3, Attack 4, and Attack 5 for the KIA SOUL and Seat Leon 2018 datasets. (**a**) Benign engine speed against generated malicious engine speed for Seat Leon 2018. (**b**) Benign engine speed against generated malicious engine speed for KIA SOUL.

To test that our framework can detect unknown attacks, some batches of real data are manipulated by applying the testing attack models mentioned in Table 2 used to generate period malicious data that have never been used before for training. Figure 8 illustrates the tuning of the engine speed PID by applying the five different attacks for the two vehicles. The attacks are applied over a particular period of time to produce semantic values, which are generating correct values according to the physical constraints of the engine speed; however, the produced values deviate the behavior from the expected one. In Attack 1, the signal during the whole time window is scaled down by a fixed value of β. In Attack 2, the signal is reduced by a different value of $\beta_{j}$ at each reading $t_{j}$ for a period of time. In Attack 3, a certain value captured at a particular time will be fixed for some time. In Attack 4, the signal values within a certain time $t_{j}$ are scaled up by a corresponding value of $\beta_{j}$. Finally, in Attack 5, the value of the signal takes the value of the previous reading multiplied by β, which differs between 0.1 to 0.9 or between 1.5 to 4 to shift and scale the normal behavior of the signal.

Attackers can manipulate, for example, engine coolant temperature by increasing its value while the vehicle operates in normal mode, which leads to operating the radiator fans in unnecessary conditions that affect engine performance in a poor way. In another scenario, if the attacker manipulates the RPM value and reduces it over its actual value, this may give a false indication to the driver to drive faster than their current speed, which could jeopardize the driver’s life.

Multiple datasets are created by leveraging the two original vehicle datasets, with the intention of incorporating malicious diagnostic parameters. The distribution of benign and malicious data within various datasets is depicted in Table 3 and Table 4. Dataset 1 serves as the training dataset for the XGBoost model in the context of known attacks. Due to its probabilistic generation, the distribution varies among different attack types. Datasets 2, 3, 4, and 5 are structured to maintain an approximately equal number of benign and malicious samples of one of the known attacks, ensuring proper training of the machine learning model and mitigating the risk of overfitting. Dataset 6 comprises both benign data and all five period anomaly attacks (classified as unknown attacks), while Dataset 7 includes benign data along with all known and unknown attacks, ensuring an equal distribution to validate the proposed framework. Datasets 8, 9, 10, 11, and 12 consist of benign data paired with individual instances of unknown attacks.

## 6. Experimental Results

The training and verification operations are simulated on an Intel(R) Xeon(R) Central Processing Unit (CPU) with two cores and a frequency of 2.30 GHz. We used detection accuracy, false acceptance rate, F1 score, precision, and detection rate (recall) metrics in the assessment of our proposed framework. The detection accuracy is calculated by obtaining the ratio of detecting the attacks and the benign data correctly to the total samples of the data:

(26) $$Accuracy=\frac{T_{N}+T_{P}}{T_{N}+T_{P}+F_{N}+F_{P}},$$

where the $T_{N}$ is the samples’ number of correctly detected benign PIDs, $T_{P}$ is the samples’ number of correctly detected malicious PIDs, $F_{N}$ is the number of samples that are malicious and detected as benign, and $F_{P}$ is the number of samples that are classified as malicious while they are benign. The false acceptance rate is determined by calculating the percentage of detecting benign values of the diagnostic parameters as anomaly data:

(27) $$FA=\frac{F_{P}}{F_{P}+T_{N}}$$

The detection rate (recall) is calculated by obtaining the ratio of the number of detected malicious PIDs to the total number of attacks:

(28) $$DR=\frac{T_{P}}{F_{N}+T_{P}}$$

The ratio of the correctly detected PIDs to total identification findings is known as precision:

(29) $$Precision=\frac{T_{P}}{F_{P}+T_{P}}$$

The F1 score is used to assess the classification model’s precision. It indicates the harmonic average of precision and recall. The balance between recall and precision is provided by the F1 score:

(30) $$F1=\frac{T_{P}}{\frac{1}{2}\left[F_{P}+F_{N}\right]+T_{P}}$$

The raw datasets of the two vehicles have been manipulated in several ways to generate different datasets to train and verify the framework. Each dataset consists of either benign PIDs alone or a mixture of benign and malicious PIDs that have been altered using known or unknown attacks. The malicious datasets may consist of a single type of attack, a combination of known attacks, a collection of unknown attacks, or a mixture of both known and unknown attacks.

**Figure 9:**
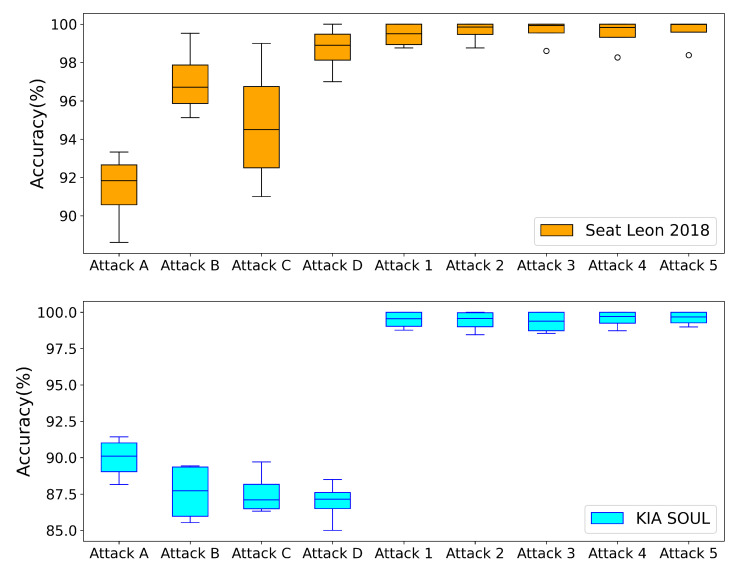
Accuracy data of GAN from several different experiments are shown in a box plot.

First, the discriminator of the GAN is tested alone against known attacks mentioned in Table 1 (using Datasets 2, 3, 4, and 5) and unknown attacks mentioned in Table 2 (using Datasets 8, 9, 10, 11, and 12), whereas it is verified against each attack separately. The accuracy results are represented in a box plot for each attack, as shown in Figure 9. From the results, GAN based on GRU is intended to identify patterns in sequential data, such as time series signals. Because it can spot the periodic pattern, it is capable of capturing periodic anomalies better, which deviate from the regular pattern that takes place at regular intervals. However, because GRUs in GANs lack the capability to recognize a single outlier in the data, they may struggle to capture random point anomalies, which are isolated deviations from the regular pattern. The model may not be able to discriminate between a random point anomaly and a valid portion of the data because it was trained on the typical pattern. As a result, in addition to GANs, other techniques such as XGBoost must be used to capture both types of anomalies.

**Figure 10:**
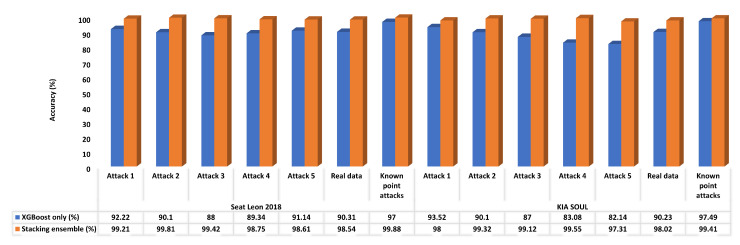
The accuracy comparison between malicious diagnostic detection framework V2 that employs the XGBoost model only [20] and the proposed malicious diagnostic detection framework V3 that has a hybrid GAN-XGBoost stacking ensemble for KIA SOUL and Seat Leon 2018 in the detection of known point attacks used in training and unknown period attacks that are not used in training.

Second, our proposed malicious diagnostic detection framework V3, which employs the hybrid GAN-XGBoost stacking ensemble, is compared against malicious diagnostic detection framework V2 [20], which employs XGBoost only as an anomaly detection model, from an accuracy perspective in the classification of unknown attacks and known attacks to highlight the performance difference between them. The XGBoost machine learning model in both versions is trained on one dataset (Dataset 1) containing a collection of known attacks (period anomaly attacks), illustrated in Table 1. Both frameworks are verified on several datasets, whereas each is composed of benign only, or benign and one of the known (Datasets 2, 3, 4, and 5) or unknown attacks (Datasets 8, 9, 10, 11, and 12). Figure 10 reflects the accuracy results of the two frameworks for the two vehicles. Usage XGBoost only in the malicious diagnostic detection framework V2 gives good results with known attacks as the model trained on those attacks; however, the modification of adding GAN provides superiority in the results of detection of the aforementioned attacks. The outcomes show that XGBoost alone cannot provide high results against unknown period anomaly attacks, while the introduced framework that deploys GAN in addition to XGBoost can detect period anomalous diagnostic data for both vehicles efficiently due to the capability of GAN in capturing the anomalous data that exceed a certain value of the reconstruction error threshold.

**Figure 11:**
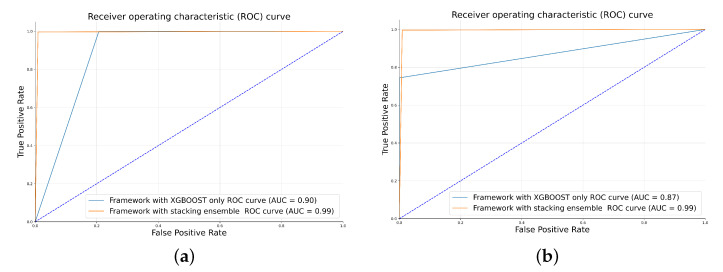
ROC curves of the malicious diagnostic detection framework V3 with the hybrid GAN-XGBoost stacked ensemble technique against the malicious diagnostic detection framework V2 with XGBoost only [20] for the Seat Leon 2018 and KIA SOUL vehicles. (**a**) ROC curve of our proposed malicious diagnostic detection framework V3 against the malicious diagnostic detection framework V2 for Seat Leon 2018. (**b**) ROC curve of the malicious diagnostic detection framework V3 against the malicious diagnostic detection framework V2 for KIA SOUL.

Figure 11 depicts the Receiver Operating Characteristic (ROC) curve and highlights the Area Under the Curve (AUC) metric of our proposed framework for different model thresholds against the malicious diagnostic detection framework V2 [20] for detecting Attack 1, Attack 2, Attack 3, Attack 4, and Attack 5 that are mentioned in Table 2. All of these attacks were merged together with real data in one dataset (Dataset 6) for each of the two vehicles to be used in the verification process.

Table 5 shows the false acceptance rate and the detection rate of our framework against the point and period attacks for the two vehicles. The framework exhibits a notable detection rate while maintaining a low false acceptance rate. This indicates a minimal rate of genuine data misclassification, reducing user distractions and confirming the framework’s efficacy. High FA can be disruptive and annoying for vehicle occupants and can erode trust in the framework, leading occupants to ignore or disable it. Furthermore, high FA triggers unnecessary responses from the vehicle’s security systems, such as locking or unlocking doors. Ensuring low FA helps maintain a safe driving environment.

The cost of each framework against its benefits can be summarized as shown in Table 6. From the results, the proposed framework has the highest cost in terms of training time and testing time (for the entire dataset); however, it has the highest accuracy and can defend against simple and complex point anomaly attacks and period anomaly attacks. The simple point anomaly attack, as described in Attack A, is exclusively represented in [33]. On the other hand, the complex point anomaly attacks mentioned in Table 1 were introduced in [20].

To roughly calculate the CPU load on a traditional automotive microcontroller unit (MCU) with a clock speed of 300 MHz (e.g., TC37x [52]), which can process 1937 frames per second (in the case of the KIA SOUL dataset), using all available CPU processing time, we can estimate that if the malicious diagnostic detection framework V3 needs to handle 200 PIDs per second, the CPU load consumed would be approximately 10%. On the other hand, the malicious diagnostic detection framework V2, being a lightweight version, would consume roughly 2% of the CPU load. Thus, in conclusion, if the need of the manufacturer for security has been limited to detecting simple and complex point anomaly attacks with reasonable intermediate training time (based on the power limitations of the platform used for running) and low CPU load, then the detection framework V2 should be employed.

Based on the analysis of the busload for all versions of the framework, it has been determined that each PID necessitates two messages: a request message and a response message. These messages are time-consuming, taking 400 μs in total due to the fact that a single CAN message requires 200 μs at a standard CAN baud rate of 500 kbps. Considering a scenario where the system needs to process 200 PIDs, it will be required to send and receive 200 CAN messages, resulting in a total time of 80 ms. Assuming the system examines all PIDs once per second, the busload in this case will be under 10%. Thus, the request messages can be issued and the responses processed by our framework during the normal operation of the vehicle (as not necessary in the parking mode) and this can be completed through the configuration of the software diagnostic stack on the ECU. It is noted that the diagnostic messages have high priority, so the framework does not send the frames back-to-back in order not to cause high jitters for other normal frames. Instead, those diagnostic frames are distributed over equal intervals. By prioritizing the most crucial PIDs and increasing their reading frequency while decreasing the rate at which less important PIDs are read, it is possible to minimize the overall busload.

**Figure 12:**
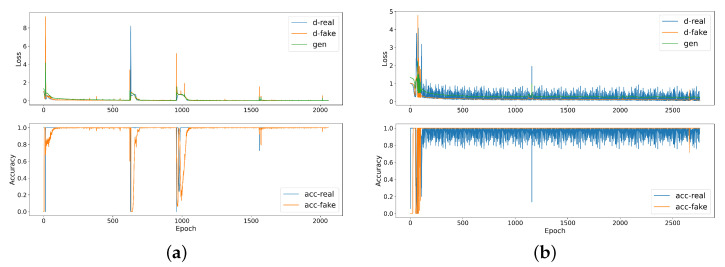
Plotting the loss and accuracy history of discriminator versus the generator through the GAN training for the two vehicles. (**a**) The loss and accuracy history of generator and discriminator in the detection of real and fake data during the GAN training for Seat Leon 2018. (**b**) The loss and accuracy history of generator and discriminator in the detection of real and fake data during the GAN training for KIA SOUL.

**Figure 13:**
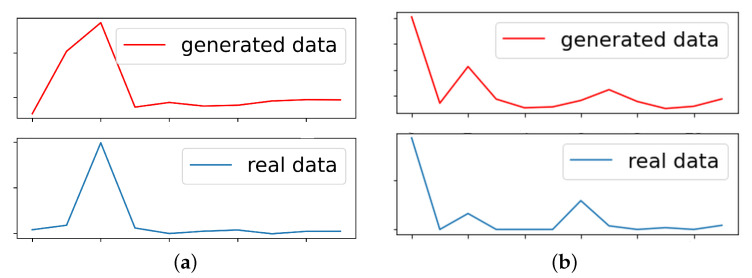
Comparing one of the signals of real data with its corresponding generated signal from the generator model of both vehicles at later stages of GAN training. (**a**) Plotting the real normalized engine speed signal of Seat Leon 2018 versus the generated signal from generator. (**b**) Plotting the real normalized fuel consumption signal of KIA SOUL versus the generated signal from generator.

The drawback of the training time of the proposed framework occurred due to training several models and the GAN’s training is the main bottleneck of the process, as it takes the highest portion of time to converge. Figure 12 depicts the loss and accuracy graph of GAN training in both vehicles. The figure shows the convergence of the generator and discriminator during training, whereas both models try to minimize the loss and increase the accuracy, and achieving the network stabilization could be quite hard requiring a lot of epochs. Figure 13 depicts a snapshot for normalized signals as an example to compare between the real signal and generated signal in order to show that the GAN has the ability to generate a fake signal that takes the same distribution of the real data for both vehicles.

Third, most of the presented IDSs focus on protecting ECUs from malicious messages over the CAN bus, which is not our perspective. Furthermore, the introduced IDS in [41] detects abnormal diagnostic sequences over CAN bus, which uses a different dataset format that is not used by our framework over CAN bus. Consequently, we applied most of the approaches mentioned in the literature besides to some of the well-known machine learning and deep learning models on datasets used in this paper to compare against the proposed framework. The proposed framework is compared against several techniques, such as random forest, decision trees, isolation forest, One Class Support Vector Machine (OCSVM), naive Bayes, CNN, Multiple-Layer Perceptron neural network (MLP) architecture mentioned in [40], GRU, and LSTM architecture mentioned in [35], ensemble approach of two classifiers and one classifier suggested by [34], a hybrid approach of CNN-LSTM introduced by [39], and the cascaded architecture compromised of DNN and GAN based on CNN proposed by [16], known as GIDS. The comparison was made with respect to the F1 score, recall, precision, and accuracy metrics. The supervised models are trained on a dataset (Dataset 1) containing benign and point anomaly attacks (known attacks), and all the IDSs are tested against another dataset (Dataset 7) that has benign data, point anomaly attacks, and period anomaly attacks (unknown attacks). Table 7 shows the F1 score, recall (R), precision (PR), and accuracy of the proposed framework against the accuracy of other aforementioned approaches for both vehicles. From the results, the random forest classifier provides the second-highest accuracy, showing its ability to predict correctly the tabular data. However, to obtain such accuracy, the number of trees should be at least 200 trees, and it is less stable than XGBoost used in the proposed framework on higher dimensional data. Naive Bayes gives poor results, as it depends on the assumption of features’ independence, which is not satisfying to detect a deviation in time series. The isolation forest relies on separating anomalies in the early stages; however, it cannot achieve a good result for both datasets, as the anomalies are hard to be separable from the benign data points in time series data. Likewise, OCSVM cannot find easily a suitable boundary to differentiate between anomalies and normal points in time series as in our case since the data are not linearly separable. As for ensemble two-class and one-class classifiers [34], it performs voting on predictions produced from ensemble two-class classifiers, which are naive Bayes, random forest, decision trees, a mixture of Gaussians, and Support Vector Machine (SVM), and predictions generated from the one-class classifiers, which are OCSVM, Mahalanobis, and support vector data description, and extreme value methods. The approach gives reasonable results as it combines the strength of each classifier; however, the convergence of one-class classifiers is not stable and takes much more time on nonlinear time series data. The accuracy of DNN models has been degraded due to the disability in the detection of period anomaly attacks that are not used in their supervising training. The hybrid approach of CNN-LSTM suggested by [39] gives higher accuracy than using alone CNN architecture or LSTM architecture as both models complement each other by effectively extracting both spatial and temporal features, leading to improved accuracy. As for IDS that depends on cascading discriminators (GIDS) based on CNN [16], it cannot provide the expected accuracy due to the inability of generating a good representation of the normal data for nonstationary data, and the data may have complex patterns and dynamics. On the other hand, the accuracy of our proposed framework is stable and overpasses all other approaches for the two vehicles.

**Figure 14:**
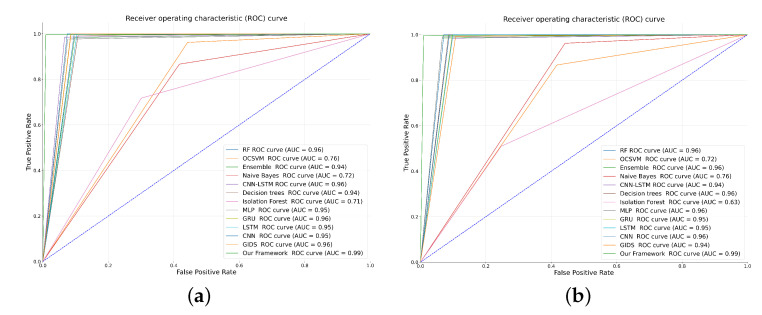
ROC curves of our proposed framework against the other machine learning models, including IDSs mentioned in the literature, verified on the KIA SOUL and Seat Leon 2018 datasets. (**a**) ROC curve of our proposed framework against the other machine learning models for Seat Leon 2018. (**b**) ROC curve of our proposed framework against the other machine learning models for KIA SOUL.

Although some approaches, such as decision trees, random forest, GRU, LSTM, MLP, CNN, CNN-LSTM, and GIDS, give reasonable accuracy, they achieve low precision, which indicates that they have a high probability error of false-positive detection. While the introduced framework attains high precision achieving a low probability error of false-positive detection, which manifests that legitimate frames are not misclassified as malicious. As for the recall measurement, it is calculated by getting the ratio of correct identification to the total number of identifications, which refers to the detection rate in our case. Based on the interpreted results, the introduced framework achieves high recall, indicating its effectiveness in identifying a significant proportion of actual attacks, which measures the system’s ability to capture true positives (i.e., correctly detected attacks) while minimizing false negatives (i.e., undetected attacks). The classification performance of the proposed framework in comparison to other models is shown in Figure 14. The superior results of the framework are gained due to the advantage of stacking and combining different strong chosen models in an ensemble to achieve the best results, where each model makes a substantial contribution to detecting the unknown and known attacks. XGBoost excels in supervised learning and effective feature engineering, while GANs, known for modeling complex data distributions, complement this by capturing subtle patterns in normal and anomalous behavior. GANs handle nonlinear relationships and uncover hidden patterns, making them adept at high-dimensional, nonlinear, and unstructured data. Ensembling allows the model to adapt to different types of data and anomalies. The XGBoost model can excel at recognizing structured anomalies, while the GAN can be more adaptive to unstructured or novel anomalies. Consequently, combining multiple models, such as XGBoost and GANs, can increase the robustness of the ensemble.

## 7. Discussion

The advantages of our framework may be summarized as follows. Because of its placement in the OSI model’s application layer, it can deal with any diagnostic protocol, making it a general framework. The proposed framework will be situated in a centralized location to avoid the requirement for extra ECUs in distributed systems. To avert a system’s single point of failure, our suggested framework can be supplemented by another powerful ECU. Nevertheless, the choice of whether to make a trade-off between the cost of additional potent ECU and the requirement of vehicle safety and security is up to the manufacturer. The bus load on the in-vehicle network buses is reduced, and there is less of a heavy demand on a specific bus or ECU, by dispersing the messages on many communication buses to query various PIDs. Without changing the core design of the in-vehicle network topology, the framework can recognize any ECU attack by spotting any anomalous change in PIDs that are not necessarily processed by this ECU but are correlated and associated with its signals. It can monitor the state of the vehicle on a regular basis during checking diagnostic updates (e.g., firmware updates), diagnostic testing, or during normal operations of the vehicle. Due to the framework’s generality, it gives the manufacturer the freedom to specify which relevant diagnostic parameters may be examined. Because the first step of the IDS exists, the detection process of malicious diagnostic data through a specification-based system is accelerated and increased. The framework is tested using data from two separate car models, one of which includes ten drivers’ driving patterns, demonstrating its stability and robustness.

Nevertheless, there are several issues that will need more attention in future work. Increasing the age of the vehicle could alter the properties of some signals. By conducting training throughout the maintenance period, the issue of vehicle aging might be resolved to reduce false alarms. The behavior of vehicle modules may vary as a result of routine maintenance and upcoming software updates, which could have an impact on the framework’s accuracy. Our framework might be retrained to address this problem, but doing so will make it vulnerable to attacks.

The primary objective of the proposed framework is to monitor the state of the vehicle and promptly report any suspicious behavior, thereby affording flexibility to users such as OEMs or system integrators to undertake appropriate actions in response to alarms. Nevertheless, it is crucial to highlight that this system does not include mechanisms for preventing attacks.

## 8. Conclusions

Our study proposes a multistage framework for detecting different anomalous cyber-physical attacks in vehicle diagnostics without increasing the busload. The framework can operate with different diagnostic protocols. It is divided into multiple stages: the first is specific-based detection and the second stage combines the XGBoost model and GRU GAN model to generate probabilities for each decision. The last stage contains a light random forest classifier to take the final decision. Then DTC is examined when anomalous behavior is detected in the diagnostic parameter, and an alarm is triggered if no DTC is present. The KIA SOUL and Seat Leon 2018 datasets are used to validate the model. We manipulated the benign data to generate point malicious data for training the XGBoost and to generate the period anomalous data to be used as unknown attacks to test our framework. The introduced framework shows the capability and performance to detect unknown period anomaly attacks and point anomaly attacks using a stacking ensemble better than using XGBoost only. Furthermore, the framework was compared against different machine learning and deep learning models and showed its superior performance that surpasses them. The results show that our framework achieves high accuracy of 99.21%, a detection rate of 99.63%, and a false acceptance rate of 0.003% for Seat Leon 2018 and an accuracy of 99.22%, a detection rate of 98.59%, and a false acceptance rate of 0.005% for KIA SOUL. In future work, the framework will be implemented and realized on the hardware level to accelerate and handle more complex functionalities. Furthermore, the framework will be trained on larger datasets and several attacks. Furthermore, it is imperative to ensure the security of the framework against unauthorized access or manipulation. Therefore, future considerations should encompass enhancing the framework’s robustness, including the employed machine learning models in this regard.

## Figures and Tables

**Table 1:** Attack models for training the anomaly detection part of the proposed framework.

Attack ID	Formula
**Attack A**	(13) $$\begin{array}{c} f(t_{l},z_{n})=f\left(t_{l},z_{n}\right)\beta ,\;ifp>0.5,0.1\leq \beta \leq 0.9 \end{array}$$ (14) $$\begin{array}{c} -f(t_{l},z_{n})\beta ,\;otherwise,0.1\leq \beta \leq 0.9 \end{array}$$
**Attack B**	(15) $$\begin{array}{c} f(t_{l},z_{n})=f\left(t_{l-1},z_{n}\right)\beta ,\;ifp>0.5,0.1\leq \beta \leq 0.9 \end{array}$$ (16) $$\begin{array}{c} f(t_{l-1},z_{n})\beta ,\;otherwise,1.5\leq \beta \leq 4 \end{array}$$
**Attack C**	(17) $f\left(t_{l},z_{n}\right)=0,\;\forall_{n}$
**Attack D**	(18) $$\begin{array}{c} f(t_{l},z_{n})=f\left(t_{l},z_{n}\right)\alpha ,\;0.1\leq \alpha \leq 0.9 \end{array}$$ (19) $$\begin{array}{c} f(t_{l},z_{m})=f\left(t_{l},z_{m}\right)\beta ,\;1.5\leq \beta \leq 4 \end{array}$$

**Table 2:** Attack models for testing the proposed framework.

Attack ID	Formula
**Attack 1**	(20) $f\left(t_{j},z_{i}\right)=f\left(t_{j},z_{i}\right)\beta ,\;\forall_{j},0.1\leq \beta \leq 0.9$
**Attack 2**	(21) $f\left(t_{j},z_{i}\right)=f\left(t_{j},z_{i}\right)\beta_{j},\;\forall_{j},0.1\leq \beta_{j}\leq 0.9$
**Attack 3**	(22) $f(t_{j},z_{i})=C,$
**Attack 4**	(23) $f\left(t_{j},z_{i}\right)=f\left(t_{j},z_{i}\right)\beta_{j},\;\forall_{j},1.5\leq \beta_{j}\leq 4$
**Attack 5**	(24) $$\begin{array}{c} f(t_{j},z_{i})=f\left(t_{j-1},z_{i}\right)\beta ,\;ifp>0.5,\forall_{j},0.1\leq \beta \leq 0.9 \end{array}$$ (25) $$\begin{array}{c} f\left(t_{j-1},z_{i}\right)\beta ,\;otherwise,\forall_{j},1.5\leq \beta \leq 4 \end{array}$$

**Table 3:** The benign and malicious distribution for each dataset for the Seat Leon 2018 vehicle.

Dataset	Benign	Attack A	Attack B	Attack C	Attack D	Attack 1	Attack 2	Attack 3	Attack 4	Attack 5
**Dataset 1**	26,469	9427	3790	5638	7613	-	-	-	-	-
**Dataset 2**	26,469	26,468	-	-	-	-	-	-	-	-
**Dataset 3**	26,469	-	26,468	-	-	-	-	-	-	-
**Dataset 4**	26,469	-	-	26,468	-	-	-	-	-	-
**Dataset 5**	26,469	-	-	-	26,468	-	-	-	-	-
**Dataset 6**	26,469	-	-	-	-	5293	5293	5293	5293	5294
**Dataset 7**	26,468	2941	2941	2941	2941	2941	2941	2941	2941	2941
**Dataset 8**	26,468	-	-	-	-	26,468	-	-	-	-
**Dataset 9**	26,468	-	-	-	-	-	26,468	-	-	-
**Dataset 10**	26,468	-	-	-	-	-	-	26,468	-	-
**Dataset 11**	26,468	-	-	-	-	-	-	-	26,468	-
**Dataset 12**	26,468	-	-	-	-	-	-	-	-	26,468

**Table 4:** The benign and malicious distribution for each dataset for the KIA SOUL vehicle.

Dataset	Benign	Attack A	Attack B	Attack C	Attack D	Attack 1	Attack 2	Attack 3	Attack 4	Attack 5
**Dataset 1**	47,212	16,703	6710	10,214	13,562	-	-	-	-	-
**Dataset 2**	47,201	47,200	-	-	-	-	-	-	-	-
**Dataset 3**	47,201	-	47,200	-	-	-	-	-	-	-
**Dataset 4**	47,201	-	-	47,200	-	-	-	-	-	-
**Dataset 5**	47,201	-	-	-	47,200	-	-	-	-	-
**Dataset 6**	47,201	-	-	-	-	9440	9440	9440	9440	9440
**Dataset 7**	47,200	5244	5244	5244	5244	5244	5244	5244	5244	5244
**Dataset 8**	47,200	-	-	-	-	47,200	-	-	-	-
**Dataset 9**	47,200	-	-	-	-	-	47,200	-	-	-
**Dataset 10**	47,200	-	-	-	-	-	-	47,200	-	-
**Dataset 11**	47,200	-	-	-	-	-	-	-	47,200	-
**Dataset 12**	47,200	-	-	-	-	-	-	-	-	47,200

**Table 5:** The false acceptance rate and the detection rate of our proposed framework for the KIA SOUL and Seat Leon 2018 datasets.

Vehicle Type	DR (%)	FA (%)
**Seat Leon 2018**	99.63	0.003
**KIA SOUL**	98.59	0.005

**Table 6:** Cost versus benefits for both frameworks.

Cost	Dataset	Framework V2 [20]	Framework V3
**Training time (s)**	Seat Leon 2018	8.94	3683.64
	KIA SOUL	71.01	29,668.50
**Testing time (s)**	Seat Leon 2018	0.38	36.98
	KIA SOUL	0.99	324.13
**Attack type**	Simple point anomaly	🗸	🗸
	Complex point anomaly	🗸	🗸
	Period anomaly	-	🗸

**Table 7:** The performance of the proposed framework for the KIA SOUL and Seat Leon 2018 datasets versus other approaches evaluated on benign, point anomaly attack, and period anomaly attack.

Model	Seat Leon 2018 (%)				KIA SOUL (%)			
	**ACC**	**PR**	**R**	**F1**	**ACC**	**PR**	**R**	**F1**
**Decision tree**	91.81	75.98	98.93	86.10	92.08	50.55	99.90	67.15
**Isolation forest**	71.54	94.61	71.76	81.62	52.14	97.60	50.96	66.96
**Random forest**	94.0	83.76	98.90	91.14	94.28	59.38	99.9	74.50
**Naive Bayes**	63.89	33.86	98.44	87.44	60.37	21.77	96.22	35.51
**OCSVM**	69.37	21.77	96.22	35.51	63.89	33.86	86.69	48.70
**Ensemble [34]**	92.45	78.66	98.44	87.44	93.87	62.59	98.55	76.56
**GRU**	92.81	69.39	99.56	81.78	90.58	40.28	99.17	57.29
**LSTM [35]**	91.05	61.59	99.90	76.21	90.39	40.25	99.90	59.63
**CNN**	91.83	65.14	99.60	78.78	91.56	34.57	99.95	51.37
**MLP [40]**	93.33	73.07	97.70	83.61	93.69	51.06	99.17	67.41
**CNN-LSTM [39]**	93.38	62.59	98.55	76.56	93.87	78.66	98.44	87.44
**GIDS [16]**	92.08	50.55	99.90	67.15	91.81	75.98	99.35	86.10
**Our framework**	99.21	96.93	99.63	98.26	99.22	96.52	98.59	97.54

## Data Availability

The links of the raw datasets have been shared in the manuscript in the References section.

## Abbreviations

The following abbreviations are used in this manuscript:

ABS	Anti-lock Braking System
ACF	Autocorrelation Function
ADAS	Advanced Driver Assistance system
AUC	Area Under the Curve
AV	Autonomous Vehicle
CAID	Context-Aware Intrusion Detection
CNN	Convolutional Neural Network
DNN	Deep Neural Network
DTC	Diagnostic Trouble Code
ECU	Electronic Control Unit
FA	False Acceptance Rate
FOTA	Firmware Updates Over the Air
GA	Genetic Algorithm
GAN	Generative Adversarial Network
GRU	Gated Recurrent Unit
HCRL	Hacking and Countermeasure Research Lab
HMM	Hidden Markov Model
HTM	Hierarchical Temporal Memory
IDSs	Intrusion Detection Systems
KIT	Karlsruhe Institute of Technology
RKE	Remote Keyless Entry
LSTM	Long Short-Term Memory
MCU	Microcontroller Unit
MLP	Multilayer Perceptron
NLP	Natural Language Processing
NN	Neural Networks
NSGA	Nondominated Sorting Genetic Algorithm
OBD	On-Board Diagnostic
OCSVM	One-Class Support Vector Machine
OSI	Open System Interconnection
OEM	Original Equipment Manufacturer
PID	Parameter Identifiers
ReLU	Rectified linear Unit
RF	Random Forest
RNN	Recurrent Neural Network
ROC	Receiver Operating Characteristic
SoC	State-of-Charge
SID	Service Identifier
SVM	Support Vector Machine
TCU	Telematics Control Unit
UDS	Universal Diagnostic Services
V2I	Vehicle to Infrastructure
V2V	Vehicle to Vehicle
V2X	Vehicle-to-Everything
XGBoost	Extreme Gradient Boosting
